# Retraction: Ten-year medication-free remission of type 2 diabetes in a South Asian male using a culturally adapted low-carbohydrate diet: an N-of-1 longitudinal study

**DOI:** 10.3389/fnut.2026.1985047

**Published:** 2026-09-07

**Authors:** Frontiers Editorial Office

The Journal retracts the 25 February 2026 article cited above.

Following publication, concerns were raised regarding authorship approval, the accuracy of author affiliations and contributions, and undisclosed conflicts of interest. An investigation, conducted in accordance with Frontiers' policies, confirmed these concerns and found that they undermined the integrity of the peer review process and the confidence in the article. As a result, the article does not meet the standards of the journal, and its conclusions have been deemed unreliable. In adherence to the recommendations of the Committee on Publication Ethics (COPE), the article is retracted.

This retraction was approved by the Chief Editors of Frontiers in Nutrition and the Chief Executive Editor of Frontiers. The authors received a communication regarding the retraction and had a chance to respond. This communication is recorded by the publisher.

